# Supplementary material for: Spatially distributed infection increases viral load in a computational model of SARS-CoV-2 lung infection
Source: PLoS Comput Biol. 2021 Dec 23;17(12):e1009735. doi: 10.1371/journal.pcbi.1009735 (PMC8740970; doi:10.1371/journal.pcbi.1009735)
Supplement: Attachment — Submitted filename: PloSCompBioSIMCoVReviewerResponse.pdf [file pcbi.1009735.s002.pdf]

Dear editors,

Thank you for the helpful reviews and the opportunity to revise and resubmit our manuscript "Spatially distributed infection increases viral load in a computational model of SARS-CoV-2 lung infection." We have addressed all of the reviewers' comments, and we believe that the paper is strengthened as a result. Below we summarize the major changes we made to the manuscript, followed by a point by point response to each reviewer comment. Attached is a version of the manuscript with changes highlighted in blue and a clean copy of the revised text.

Best regards,

Melanie E. Moses

Professor of Computer Science, University of New Mexico

(On behalf of all co-authors)

Summary of Major Revisions

- We replicated (10 to 30 times) each run in Figures 3, 4 and 5 with a different random seed each time and uniform random placement of the FOI in response to comments from all reviewers.
- In Figure 4 we added a panel to show the effect of varying the viral clearance term (in addition to T cell production and delay) since the clearance parameter is varied (slightly) to match patient data as requested by Reviewer 1.
- We added a new Figure (Fig. 8) to show a sensitivity analysis of the effect of one-at-a-time variation of 12 parameters on peak viral load and % of cells infected as requested by Reviewer 3.
- We reorganized the discussion to summarize main findings, compare to other spatial models and list limitations and future work.
- In Materials and Methods we added a section to explain how manual fits were achieved; we also added a description of the ODE model we used to compare to patient data; and we clarified how several parameters were derived as requested by Reviewer 2.
- We added several references and made minor clarifications in the text.

Specific responses to each reviewer comment

Reviewer #1

Overall thoughts:
In the article titled “Spatially distributed infection increases viral load in a computational model of SARS-CoV-2 lung infection”, the authors present an ABM model – SIMCoV – that replicates viral and immune cell dynamics following SARS-CoV-2 infection on either a 2D or 3D representation of the lung. This model can recapitulate patient specific data as well as other known phenomenon associated with SARS-CoV-2 infection. The authors provide a compelling argument: that variability in viral loads may be due to FOI and T cell magnitude/timing. Additionally, the authors provide comments and comparison between modeling techniques, an extension of the work that may be particularly applicable to other fields/simulation in lung pathology. The manuscript is well-written and clearly conveys the results. I recommend acceptance with minor revision, with comments listed below.

We thank the reviewer for this assessment and respond to recommended revisions below.

Major comments:
1. At the time of review, https://github.com/AdaptiveComputationLab/simcov is a broken link. Please ensure there is a useable link.
We have corrected this and made the link public.

2. The Results section titled: ‘Peak viral load is proportional to the number of initial FOI’ provides an intriguing result comparing the number of FOI to the number of virions w/in the simulation. While the peak differences (Fig 3 c & d) appear significant, there is a lack of statistical comparisons
We have modified the figure to show a uniform random placement of FOI so that each run varies depending on where each FOI is placed. We now show the minor variation and the standard deviations for each simulation. The (very small) differences between runs are now more clear with statistical comparisons included.

and Fig 3b suggests that all simulations sterilize infection between day 20 and 22. Please provide some greater context to this finding - e.g., is a difference of two days meaningful in the context of infection outcomes?
This happens to be a combination of parameters that leads to viral clearance in all cases. That difference of two days is not something we think is meaningful. Figure 3 is meant to highlight the linear relationship between the number of FOI and the peak viral load. We focus on how the T cell response affects the timing and ability to control infection in Figure 4, so clearance of infection is analyzed in that figure.

Additionally, perhaps there are regions of parameter space where a large FOI and a delayed T-cell response result in a total inability to control infection, whereas a small FOI even w/ delayed T-cell response can result in controlled infection. Later, the authors indirectly present this argument by comparing to patient data, but a more explicit exploration of this parameter space (small/Large FOI + large/small T cell generation) could strengthen the argument that FOI and T cells together explain variability from patient to patient.

This is a good question which we now highlight in the analysis of Patient A. That model fit shows how a very large number of FOI (220) leads to lack of control by even a large T cell response (200K T cells/min produced beginning on day 8) and default clearance parameters.

Minor comments:

1. Page 4 (first paragraph) – several modeling assumptions were not backed with references. As this is the results section, simply adding a reference will do. Example: "After seven days (ref why 7 days is appropriate -ref 40 perhaps?), we create an abstract pool representing T cells that are activated in lymph nodes and then circulate in the vasculature. When T cells reach the lung and encounter a concentration of inflammatory signal above a threshold (whats the threshold, and why is it a reasonable choice), they …"

The Materials and Methods section contains references and explanations for how each parameter is derived in detail. We removed the reference to 7 days from this introductory paragraph because that is a default parameter that is varied in several experiments. The parameter table has the appropriate references. The last sentence of this paragraph points to that more detailed explanation in Materials and Methods.

2. In Materials and Methods, please explain the decision for T-cell movement to be random within the tissue instead of chemokine-dependent (using inflammatory signals as a proxy).

In our model T cells extravasate based on the presence of inflammatory signal above a threshold, but once extravasated, they move randomly. (This is a difference from the Levin et al model in which T cells followed a chemokine gradient in the tissue.) We agree that T cell movement patterns are interesting and we have investigated T cell movement patterns in prior work. However, it is not clear how important chemokine dependent movement is for extravasated T cells in the lung. Ariotti et al described movement mediated *by CXCR3* as a subtle but important effect, and in our work (Mrass et al 2017) we saw little change in T cell movement in the lung when chemokine receptor signaling was blocked. We included in Discussion that a more detailed analysis of T cell movement is left for future work. We intend to pursue this in a forthcoming version of SIMCoV with a more mature model of lung structure.

3. Figure Captions should provide more information. For example, Figure 3b FOI_1_1 and FOI_1_16 should be explained in the caption.

We have clarified this in the caption in Figure 3.

4. I appreciated the thorough explanations within the Parameter Derivation section. One brief comment about k and l. Intrinsically, these production and decay parameters represent these processes for many unique cytokines that have been grouped together as 'inflammatory signals'. This explains why there are not explicit measurements of these parameters in the literature, and this should be mentioned within this section. As a sanity check, one could compare these estimated values against known inflammatory cytokines (such as TNF).

The reviewer is correct that the parameter is difficult to estimate for that reason. We added this explanation and refer to the production rates from the Levin et al model and Mitchell et al experimental paper that estimates production for IP-10 and RANTES for influenza. We note in the discussion the uncertainty in the inflammatory parameters, but that the last row of Table 8 also shows that the model is quite insensitive to these parameters.

5. In the discussion, please comment on the modeling decision to exclude other T cell phenotypes such as CD4+ T cells or regulatory T cells which are both thought to potentially play a role in COVID-19 outcomes (https://www.nature.com/articles/s41577-020-0402-6#Sec4).
We now explain more clearly that we focus specifically on CTLs that directly kill virus-infected cells.

6. In table 2, include an upper/lower bound for the parameters that were varied in order to capture the patient data in Fig 5.

We have addressed this point in the new paragraphs explaining the manual fitting procedure in the subsection "Fitting to Patient Data" in the Methods and we highlight the bounds of the fitting in the main text.

Reviewer #2: In this study, the authors present a large-scale agent-based model to simulate and investigate infection and immune dynamics in lung epithelial tissue during SARS-CoV-2 infection. The Spatial Immune Model of Coronavirus (SIMCoV) considers the dynamics of lung epithelial cells, SARS-CoV-2 viral load, inflammation signals and CD8+ T cells within a spatio-temporal context. Analyzing their modeling environment they find that heterogeneity in patient outcomes could be explained by the number of initial fields of infection within lung tissue, as well as the timing of the T cell response.

The article is generally well written and structured. However, some aspects regarding the analyses and methodology are only insufficiently explained and would need some further explanations. This especially relates to the parameterization of the agent-based model, which is a key aspect for such highly complex models and its comparison to patient data. In addition, the analysis seems a bit limited in terms of making use of the stochasticity that an ABM, especially one considering the spatial distribution of cells, can provide.

We thank the reviewer for these helpful comments and suggestions and have improved our analysis of model stochasticity and further explained model parameterization as we explain below.

Major points:

(1.) SIMCoV represents an extended agent-based modeling environment to simulate and study viral infection within tissue in a spatially defined context. It is claimed that considering infection dynamics in a spatially-resolved context helps to explain the variation observed in individual patient outcome to SARS-CoV-2 infection, with the number of initially infected sites being an

important factor determining disease progression. To which extent could this not also be covered by different initial viral loads within an ODE-model, as well as different timing of the T cell responses?

We now emphasize more clearly in Figure 3 that the initial viral load per se has little impact on peak viral load (compare FOI_16_1 to FOI_1_1). However, that same figure shows that if the viral load is spread among multiple sites, the peak increases linearly with the number of FOI. We do not know how an ODE that makes an assumption of well-mixed interactions could capture this inherently spatial effect. SIMCoV is able to distinguish between the *amount* of initial inhaled virus (which does not directly impact the peak viral load), and also how that initial amount of virus is distributed in space (which substantially impacts the peak viral load). SIMCoV suggests that the initial inhaled virus could lead to different outcomes, depending on the spatial distribution of that load in different FOI. We note in the Discussion that several ODE modeling papers have incorporated T cell response as a generic increase in the clearance term, and PDEs have considered diffusion and advection, but the variation in FOI is an inherently spatial effect.

While it is investigated how the distribution of the same number of infected cells within different numbers of initial fields of infection influence the dynamics, I could not follow the claim that this analysis might provide a better explanation of variation in patient outcome than the analysis provided by the ODE model. Both seem to explain the data comparably well, judged from the RMSE (see also point 3).

The reviewer is correct. We did not intend to claim that the SIMCoV fits are closer to the data than the ODE fits; they are indeed similar. Our claim is that we are able to fit the data by modifying only a few key parameters that represent biologically plausible mechanisms that affect peak viral load and ability to clear infection. For example, we hypothesized that more FOI would lead to a higher peak viral load, and we were able to show that both in Figure 3 and in fits to the data in Figure 5. We changed only the number of FOI, the T cell generation rate and timing and the clearance term (over a small range) to fit the data. This is explained in the new Materials and Methods “Fitting Patient Data” section.

(2.) Agent-based models usually provide stochastic outputs given repeated simulations with the same parameter set. However, in each of the figures (e.g. Figure 3,4,5,7) only a single line for the model simulations of a particular scenario is shown. It is not clear or specified within the figure legends if this is the mean over several simulation runs or a single representation. In any case, it would be very important to know how much variation in the dynamics can occur from one given model parameterization. Especially the distribution of the initial fields of infection will potentially have a substantial impact on the observed dynamics in such a spatial modeling environment. In this regard, the authors should investigate how non-equidistant distribution of FOI (see Fig. 3a) would affect the model outcome and variation. The authors need to report the variation in model outcomes to corroborate their claims, i.e. that the initial number of FOI partly determines disease outcome.

We follow the reviewer's excellent suggestion in the new Figure 3 to analyze how FOI placed at uniform random affect the peak viral load. We replicate the model 30 times with 30 different random placements. This introduces a small amount of variation (visible on the log scale only at the

end of the runs with small viral loads where variation is more visible). The linear relationship between number of FOI and peak viral load remains. We also now also show multiple runs of the model in Figs 4 and 5.

(3.) Figure 5: How was SIMCoV adapted to the patient data? On page 6, bottom, the phrasing leaves the impression that SIMCoV was actually "fitted" to the data. What kind of fitting procedure was used for this task? From the Materials and Methods section, I get more the impression that several parameters were varied and manually adapted according to values and parameter ranges from the literature. But it is not clear which parameters were fixed and which ones were varied/fitted/adapted. If only the initial FOI was varied to explain the patient data in Figure 5 (p.6/7) it is also not clear if FOI were always equidistantly (as shown in Figure 3a) or randomly placed within the grid. If several parameters were varied, are the parameters identifiable or are they correlated given the complexity of the model? If the simulations were manually adapted, the authors might want to consider automatic parameter inference methods, such as approximate Bayesian computation (ABC-methods), as they have been used before for agent-based models to analyze multi-cellular systems and infection dynamics (e.g. Jagiella et al. Cell Systems 2017, Imle et al. Nat Com. 2019). Parameterizations of such modeling environments is one of the key aspects for obtaining reliable predictions and simulations.

We removed the confusing wording that implied that the parameters were fit automatically. The manual fitting is explained in the new Materials and Methods "Fitting Patient Data" section. We now show random placements of FOI in Figure 3 rather than uniform placements (although that does not change the outcome much.) Our approach to fitting was not the same as the statistical fitting approaches that are often used to fit parameters in ODE models. We instead varied only parameters that seemed likely to vary among patients. Our goal was to demonstrate the plausibility of the biological mechanisms, namely that the number of FOI could explain the viral peak and the timing and magnitude of T cell response could explain the shape of the decline.

(4.) Some details on how the ABM was implemented are missing. While the different processes are explained and introduced, it is not fully specified how these processes are actually parameterized, i.e. how probabilities of events were calculated and simulated. While it is very applaudable that the authors make their simulation environment publicly available, appropriate details on how the parameters were used within the code seem to be missing, if I did not miss this within the text.

We have included a 4-page model description in the Materials and Methods section. We are not sure what particular probabilities are missing. We note that in most cases, parameters are constant values rather than probability distributions. The model description does specify that time periods are drawn from a Poisson distribution and infectivity is modelled as a rate (a fixed probability) with all parameters shown in Figure 1 and detailed in Table 2 and points *a* through *r* below the table.

(5.) The ODE-model used to explain the individual patient data is not introduced, nor the methods how this was fitted to the data (e.g. mixed-effect model?, individual fits?). Even though this might be published previously, I think the details have to be repeated within the manuscript to compare the different modeling approaches and evaluate the outcomes. In addition, confidence intervals should be given for the estimates within Table 1.

We have added an explanation of how the ODE was fit in the previous work in Materials and Methods. Because the estimates in Table 1 are fit by hand, we do not have CI. The parameters in Table 1 should be interpreted as plausible values that fit the data, not the values that provide an optimal fit. Our goal with the SIMCoV model is not to provide an optimal set of parameters, but to suggest biologically meaningful hypotheses about what mechanisms might produce outcomes in the viral load trajectories -- specifically that the number of FOI can linearly influence peak viral load and that the timing and magnitude of T cell response can determine the duration of infection and probability of clearance in the first month of infection.

Minor points:

- page 2, bottom: If epithelial cells are modeled as a 2D grid, it is not clear why they have to be represented as a volume. Does this play a role for e.g. diffusion?

If SIMCoV were only used to model a 2D sheet of cells, it would not be essential to represent cell volume with a z dimension. However, we also use SIMCoV to model dynamics in a 3D grid and branching topology (Figures 6 and 7) where the 3rd dimension is necessary. We plan to further investigate the role of diffusion in different topologies in future work.

- Figure 7: How comparable are the different scenarios when studied in 2D and 3D? Were the same number of cells/ target cell densities used, as well as the same number of T cells able to infiltrate the tissue?

Yes, these things were all held constant (Default parameters). We added a sentence to make that more clear.

- Figure 3d: I am not sure how much informative the peak number of T cells would be on viral control in order to compare the different settings. The peak might occur at different time points and the overall level of the T cell responses might be similar. Wouldn't the cumulative T cell concentration up to a certain time point, and potentially a ratio between T cells and Viral load be more informative for a comparison between the different scenarios, i.e, clearly indicating that the spatial distribution of the number of locations and not the magnitude of initial infection determines disease progression?

In these runs the peak and cumulative number of T cells are highly correlated. With much higher viral dispersion, we might expect to drain the supply of T cells; however we need larger simulations to see that effect. We agree that the ratio of peak virus to T cells (visible in the corresponding pattern in panels c and d) are an interesting consequence of the T cells being drawn into the lung by the space occupied by virus, and therefore releasing inflammatory signal.

- There have been other studies that introduced multi-cellular modeling environments for studying viral infections within epithelial tissues (Sego et al. PLos Comp Biol. 2020). The authors might want to discuss how their approach relates to those. In addition, the authors should discuss why their model is especially a modeling environment for coronaviruses, and not viral infections per se. Only in the last part when studying lung structure, the modeling environment seems to become lung specific.

We have now added a section to the discussion comparing SIMCoV to Sego et al 2020 and other models. We also added that the SIMCoV modelling platform can be extended to model other organs and other viruses.

Reviewer #3: In this paper, Moses et. al. propose a nicely designed mathematical model that includes spatial aspects of infection to recapitulate the SARS-CoV-2 viral load variability among infected individuals. The model is an agent-based one that simulates infection dynamics and CD8 responses in the space of millions of epithelial cells. This approach is relevant as it may explain how more heterogenous virus dynamics could be produced as a function of the spatial parameters, something that ordinarily differential equations are limited to do in this context. Below are my comments:

We thank the reviewer for this complimentary summary.

1. Regarding the peak viral load as a function of the initial FOI, I was wondering if the proportionality is also affected by spatial distribution of the virions. In other words, if the initial FOI and initial virions on them are randomly distributed in the grid—and not just equally dispersed—would the peak viral load still be proportional? What if all the initial FOI is in a specific place in the grid? In a target cell-limited model, or even those that include effector cells, the peak viral load wouldn't change as a function of the initial viral load. In that sense, it would be helpful to have a more elaborated mechanistic explanation of why this is not the case in the spatial model. Examples of this would be to have spatial simulations of infected cells and CD8 cells per each case illustrating the possible mechanisms. Is the proportionality only due to more infection sites or due to something else like the dispersion of CD8 cells that decrease the chances to attack the infection?

Figure 3 now analyzes a uniform random placement of cells and we have added text to highlight that when more virus is added to the same location, there is almost no effect on peak. It is only when more virus is added to new FOI that the peak increases. The variation shown with different placements of FOI occurs because if the locations are nearby, they merge into each other, reducing the number of newly infectable cells. This is particularly evident for larger numbers of FOI (16) with more potential for overlap in Figure 3.

2. Although authors present the effect of two parameters related to the CD8 response with respect to the virus dynamics, it would be also helpful to do a sensitivity analysis on how that compares to the effect of other parameters in Table 2. Which parameters are driving each phase of the virus dynamics? If there are multiple parameters that drive one aspect of the dynamics, which one is more significant? This is relevant in the sense that it would highlight in which aspects the spatial aspects (diffusion rates, initial distribution of variables in the grid, etc) are more significant in the virus dynamics than the non-spatial ones.

We have added Figure 8 to show how variation in 12 parameters affects peak viral load and the number of infected cells. The spatial parameters are not necessarily more significant than non-spatial parameters, but they are significant, particularly the number of FOI.

3. After having a set of estimated parameters from the model fits, it would be more convincing, in terms of viral load variability, if the model could be simulated multiple times to show that it does allow for more heterogeneity in terms of peak levels, time to peak, time to shoulder phase and time to control.

In response to this and other reviewer questions, we now show how variation in the placement of FOI and multiple runs generate variation in Figures 3, 4 and 5. Figure 8 and the discussion now also address this point.

4. It seems to me the paper is lacking a section (in the Materials and Methods) describing the fitting procedure in detail.

We have added a 2 paragraph explanation of the manual fitting procedure in the subsection "Fitting to Patient Data" in the Methods.

5. Would the model be able to reproduce multiple viral peaks as in figure 5G?

We didn't attempt to fit this aspect of that anomalous patient. In future work we are modeling more complex spatial spread of virus through the airways, in which case we might be able to account for this.

6. The github page did not work when I tried to open it.

We have fixed this error. It is now publicly available.

Have the authors made all data and (if applicable) computational code underlying the findings in their manuscript fully available?

The PLOS Data policy requires authors to make all data and code underlying the findings described in their manuscript fully available without restriction, with rare exception (please refer to the Data Availability Statement in the manuscript PDF file). The data and code should be provided as part of the manuscript or its supporting information, or deposited to a public repository. For example, in addition to summary statistics, the data points behind means, medians and variance measures should be available. If there are restrictions on publicly sharing data or code —e.g. participant privacy or use of data from a third party—those must be specified.

Reviewer #1: **No:** The link to the code repository was broken. "page not found".

We have fixed this error. It is now publicly available.

Reviewer #2: Yes

Reviewer #3: Yes

PLOS authors have the option to publish the peer review history of their article (what does this mean?). If published, this will include your full peer review and any attached files.

If you choose "no", your identity will remain anonymous but your review may still be made public.

Do you want your identity to be public for this peer review? For information about this choice, including consent withdrawal, please see our Privacy Policy.

Reviewer #1: No

Reviewer #2: No

Reviewer #3: No

Figure Files:

While revising your submission, please upload your figure files to the Preflight Analysis and Conversion Engine (PACE) digital diagnostic tool, https://pacev2.apexcovantage.com. PACE helps ensure that figures meet PLOS requirements. To use PACE, you must first register as a user. Then, login and navigate to the UPLOAD tab, where you will find detailed instructions on how to use the tool. If you encounter any issues or have any questions when using PACE, please email us at figures@plos.org.

Data Requirements:

Please note that, as a condition of publication, PLOS' data policy requires that you make available all data used to draw the conclusions outlined in your manuscript. Data must be deposited in an appropriate repository, included within the body of the manuscript, or uploaded as supporting information. This includes all numerical values that were used to generate graphs, histograms etc.. For an example in PLOS Biology see here: http://www.plosbiology.org/article/info%3Adoi%2F10.1371%2Fjournal.pbio.1001908#s5.

All instructions and configuration files to reproduce each figure are included in the github repo.

Reproducibility:

To enhance the reproducibility of your results, we recommend that you deposit your laboratory protocols in protocols.io, where a protocol can be assigned its own identifier (DOI) such that it can be cited independently in the future. Additionally, PLOS ONE offers an option to publish peer-reviewed clinical study protocols. Read more information on sharing protocols at https://plos.org/protocols?utm_medium=editorial-email&utm_source=authorletters&utm_campaign=protocols
