# Supplementary material for: Spatially distributed infection increases viral load in a computational model of SARS-CoV-2 lung infection
Source: PLoS Comput Biol. 2021 Dec 23;17(12):e1009735. doi: 10.1371/journal.pcbi.1009735 (PMC8740970; doi:10.1371/journal.pcbi.1009735)
Supplement: Attachment — Submitted filename: ResponseToReviewers2_SIMCoV.pdf [file pcbi.1009735.s003.pdf]

Dear Editors and Reviewers

We thank you for your very helpful suggestions that have improved our manuscript. We made the following changes.

We added an explanation for why the replicates of multiple FOI runs were so similar – because we avoided overlapping FOI in the large simulation size, and we avoided boundary effects by not placing FOI near the simulation edges.

We re-worded our process of manually parameterizing the model to fit patient data.

We moved Figure 8 (the sensitivity analysis) up to become Figure 5 in the Results section.

We made the suggested minor corrections and clarifications.

We have uploaded a version of the manuscript with new changes in red as well as a clean version. We have also updated Figure names and numbers to reflect the changed order.

We look forward to the next steps!
Very best regards,
Melanie Moses (on behalf of all co-authors)

Reviewer's Responses to Questions

Comments to the Authors:
Please note here if the review is uploaded as an attachment.

Reviewer #2: In this revised version, the authors have addressed all of my previous comments by performing additional analyses and extending previous explanations on the used methods within the manuscript. This clearly has improved and clarified the manuscript. However, I still would have some comments that I would be happy to see addressed by the authors.

Major points:

(1.) Figure 3b): It is quite surprising that even given random positioning of individual initial foci, the variation between different simulation runs is basically non-existent. Given the large dependency of the infection dynamics on the initial foci number this is difficult to believe. As indicated in Figure 2, given the chosen parameterization of viral diffusion and infectivity, the infection seems to be largely spreading to directly

neighboring cells, i.e. no seeding of new foci by diffusing virions. This means infection spread depends on the combined “surface area” of all foci. However, if foci were seeded randomly, I would expect that at least some foci are initiated closely together, merge early or are initialized close to the boundary of the grid, so that this effectively decreases the “spread surface”, and, thus, should lead to some variation between the simulations. Did the authors also had a look at the corresponding plot for Figure 3b for the number of infected/uninfected epithelial cells? Did this also not reveal any variation in the dynamics? At least for 16 randomly placed, initial foci, I would expect this.

We added the following text to explain why the multiple runs in Figure 3 are so consistent. "Because the simulated tissue size is large relative to the FOI, even at their peak extent, it is statistically unlikely that randomly placed FOI will grow to have any overlap in the simulations. In Figure 3, each FOI, even at its peak extent, comprises less than 0.2% of the entire simulated tissue, which in this case is 225 million cells. In addition, we located all FOI in the inner 80% of the simulated tissue to avoid boundary effects."

It is also worth noting that Figure 3a is a conceptual image, not a screenshot of the simulation. We now note in the caption that the sizes of the infected regions shown in Figure 3a are amplified so each infected location and their relative size can be visualized. (At real scales, most FOI are likely imperceptible dots below the resolution of detection). More generally, your point is well taken, and our current research is addressing questions about spatial distributions of FOI in more detail and validating those spatial features against CT scans. However, we have discovered that the issues are quite complex and beyond the scope of this paper.

(2.) Although this might be only semantic, I would recommend to speak of “adapting” the model to data rather than “fitting”. In my view, fitting always implies an automatic/algorithmic approach to minimize a difference between model and data. “Manual fitting” could be misleading in this sense. Although it is indicated that some ordered way of adjusting the parameters for each individual patient is used (novel paragraph in M&M), it is still not an elaborated search of the possible parameter space (i.e. fixed parameter ranges for all patients), as has been acknowledged by the authors.

We did not want to imply that there was any automatically adaptive feature to our procedure, but we’ve changed the wording to refer to the process as parameterizing the model, and we changed the title of the relevant section to “Manually Parameterizing SIMCoV to Fit Patient Data”.

Minor points:

- Related to point 1 above: I couldn’t find within the text if for the 2D simulations in Figure 2D and 3, periodic or fixed boundaries were used for the simulations.

We now specify in the model description: Grid boundaries are fixed, not periodic.

- p8 bottom: "… controlled by a even a high …."

Corrected

- Fig. 7: How many foci were used to start the infection in the different topologies? I would assume this should be a single infected cell to allow comparison.

This is correct, and now specified in the caption

Reviewer #3: The authors have addressed all my comments successfully.

My only comment is that the results of Figure 8 seemed a little out of place. It was odd to see results of peak viral load in CD8/viral clearance results and the methods section. My suggestion would be to divide Figure 8 in two:

-The first figure would include how parameters affect the viral peak only and include a reference to those results in the second section of the results, "Peak viral load is proportional...". In this way, it would show better how the effect of FOI in the viral peak is more prominent than the other parameters in the same section. The figure related to the viral peak could be part of Figure 3.

-The second figure would include how parameters affect the final percentage of infected cells (as a measure for viral clearance) and could be part of Figure 4. This figure would be referenced in the "Effect of CD8+ T cell response on viral clearance" section.

In this way, Figure 8 is not at the end/methods, and its results would follow the flow of the paper.

Thank you for this suggestion. We moved Figure 8 up to become the new Figure 5 and put appropriate pointers in the caption so that the percentage of infected cells makes sense in the flow of the paper.

Have the authors made all data and (if applicable) computational code underlying the findings in their manuscript fully available?
The PLOS Data policy requires authors to make all data and code underlying the findings described in their manuscript fully available without restriction, with rare exception (please refer to the Data Availability Statement in the manuscript PDF file). The data and code should be provided as part of the manuscript or its supporting information, or deposited to a public repository. For example, in addition to summary statistics, the data points behind means, medians and variance measures should be

available. If there are restrictions on publicly sharing data or code —e.g. participant privacy or use of data from a third party—those must be specified.

Reviewer #2: Yes

Reviewer #3: Yes

PLOS authors have the option to publish the peer review history of their article (what does this mean?). If published, this will include your full peer review and any attached files.

If you choose “no”, your identity will remain anonymous but your review may still be made public.

Do you want your identity to be public for this peer review? For information about this choice, including consent withdrawal, please see our Privacy Policy.

Reviewer #2: No

Reviewer #3: No
