# Supplementary material for: Spatially distributed infection increases viral load in a computational model of SARS-CoV-2 lung infection
Source: PLoS Comput Biol. 2021 Dec 23;17(12):e1009735. doi: 10.1371/journal.pcbi.1009735 (PMC8740970; doi:10.1371/journal.pcbi.1009735)
Supplement: Attachment — Submitted filename: ReviewOf_PCOMPBIOL-D-21-01181.docx [file pcbi.1009735.s001.docx]

Overall thoughts:

In the article titled “Spatially distributed infection increases viral load in a computational model of SARS-CoV-2 lung infection”, the authors present an ABM model – SIMCoV – that replicates viral and immune cell dynamics following SARS-CoV-2 infection on either a 2D or 3D representation of the lung. This model can recapitulate patient specific data as well as other known phenomenon associated with SARS-CoV-2 infection. The authors provide a compelling argument: that variability in viral loads may be due to FOI and T cell magnitude/timing. Additionally, the authors provide comments and comparison between modeling techniques, an extension of the work that may be particularly applicable to other fields/simulation in lung pathology. The manuscript is well-written and clearly conveys the results. I recommend acceptance with minor revision, with comments listed below.

Major comments:

1. At the time of review, <https://github.com/AdaptiveComputationLab/simcov> is a broken link. Please ensure there is a useable link.
2. The Results section titled: ‘Peak viral load is proportional to the number of initial FOI’ provides an intriguing result comparing the number of FOI to the number of virions w/in the simulation. While the peak differences (Fig 3 c & d) appear significant, there is a lack of statistical comparisons and Fig 3b suggests that all simulations sterilize infection between day 20 and 22. Please provide some greater context to this finding - e.g., is a difference of two days meaningful in the context of infection outcomes? Additionally, perhaps there are regions of parameter space where a large FOI and a delayed T-cell response result in a total inability to control infection, whereas a small FOI even w/ delayed T-cell response can result in controlled infection. Later, the authors indirectly present this argument by comparing to patient data, but a more explicit exploration of this parameter space (small/Large FOI + large/small T cell generation) could strengthen the argument that FOI and T cells together explain variability from patient to patient.

Minor comments:

1. Page 4 (first paragraph) – several modeling assumptions were not backed with references. As this is the results section, simply adding a reference will do. Example: “After seven days (**ref why 7 days is appropriate -ref 40 perhaps?**), we create an abstract pool representing T cells that are activated in lymph nodes and then circulate in the vasculature. When T cells reach the lung and encounter a concentration of inflammatory signal above a threshold (**whats the threshold, and why is it a reasonable choice**), they …”
2. In Materials and Methods, please explain the decision for T-cell movement to be random within the tissue instead of chemokine-dependent (using inflammatory signals as a proxy).
3. Figure Captions should provide more information. For example, Figure 3b FOI_1_1 and FOI_1_16 should be explained in the caption.
4. I appreciated the thorough explanations within the Parameter Derivation section. One brief comment about k and l. Intrinsically, these production and decay parameters represent these processes for many unique cytokines that have been grouped together as ‘inflammatory signals’. This explains why there are not explicit measurements of these parameters in the literature, and this should be mentioned within this section. As a sanity check, one could compare these estimated values against known inflammatory cytokines (such as TNF).
5. In the discussion, please comment on the modeling decision to exclude other T cell phenotypes such as CD4+ T cells or regulatory T cells which are both thought to potentially play a role in COVID-19 outcomes (<https://www.nature.com/articles/s41577-020-0402-6#Sec4>).
6. In table 2, include an upper/lower bound for the parameters that were varied in order to capture the patient data in Fig 5.
